# Non-Contact Heart Rate Detection When Face Information Is Missing during Online Learning

**DOI:** 10.3390/s20247021

**Published:** 2020-12-08

**Authors:** Kun Zheng, Kangyi Ci, Jinling Cui, Jiangping Kong, Jing Zhou

**Affiliations:** 1 Faculty of Information Technology, Beijing University of Technology, Beijing 100224, China; zhengkun@bjut.edu.cn (K.Z.); cikangyi@emails.bjut.edu.cn (K.C.); kongjp@emails.bjut.edu.cn (J.K.); 2 College of Continuing Education, Beijing University of Technology, Beijing 100224, China

**Keywords:** heart rate, non-contact, region of interest, FastICA, remote photoplethysmography

## Abstract

Research shows that physiological signals can provide objective data support for the analysis of human emotions. At present, non-contact heart rate data have been employed in the research of medicine, intelligent transportation, smart education, etc. However, it is hard to detect heart rate data using non-contact traditional methods during head rotation, especially when face information is missing in scenarios such as online teaching/learning. Traditional remote photoplethysmography (rPPG) methods require a static, full frontal face within a fixed distance for heart rate detection. These strict requirements make it impractical to measure heart rate data in real-world scenarios, as a lot of videos only partially record the subjects’ face information, such as profile, too small distance, and wearing a mask. The current algorithm aims to solve the problem of head deflections between 30 degrees and 45 degrees by employing a symmetry substitution method, which can replace the undetected region of interest (ROI) with the detectable one. When face information is partially missing, our algorithm uses face–eye location to determine ROI. The results show that the method in this paper can solve certain practical problems related to heart rate detection, with a root mean square error (RMSE) under 7.64 bpm.

## 1. Introduction

With increasing interest in non-intelligent factors in the learning process in the field of education research, people are beginning to pay attention to the emotional problems in education and teaching, especially those associated with students’ learning. As an important non-intelligence factor, emotion has a multi-dimensional and all-round influence on students’ growth and development [1]. Thus, expressing emotions through data plays an important role in intelligent education. At present, emotion recognition is mainly realized through facial expression recognition. Although the method is simple and does not need special equipment, it cannot guarantee accurate emotion recognition. On one hand, people can disguise their true feelings by affecting their facial expressions, which is often difficult to detect [2]. On the other, there are a lot of emotions that are not clearly conveyed through facial expressions. Furthermore, different personalities lead to different abilities in terms of expressing various emotions. Therefore, emotional states need to be represented in a higher dimensional space.

Research shows that physiological signals can provide objective data support for emotion recognition [3]. Thus, a relationship can be established between emotional recognition and heart rate. Bland et al. [4] evaluated the heart rate responses of students answering a series of questions related to the theory of evolution and found that students would produce perceptible emotional responses in the form of physiological changes when they were exposed to the theme of evolution. Stavroulia et al. [5] used heart rate data to evaluate the emotional changes of subjects in certain scenarios and virtual reality experiences. They found that the heart rate increased when the subjects’ moods changed significantly. In addition, when Nik et al. [6] designed an online learning guidance system that can detect and reflect learners’ emotional states, heart rate variability was selected to measure learners’ emotional changes. 

Contact heart rate estimation equipment, such as electrocardiogram (ECG) or conventional photoplethysmography (PPG), requires electrodes, gel, and experienced nurses or doctors, and usually causes skin irritation and discomfort. Moreover, it is not convenient for students to wear special instruments to measure heart rate in online classes. However, detecting heart rate through non-contact methods does not affect the students’ daily life. 

Camera-based remote photoplethysmography (rPPG) enables low-cost, non-contact cardiovascular activity testing. Online teaching is often carried out on computers or mobile phones. These electronic devices with cameras can be used to collect students’ physiological signals. 

Video-based physiological signal detection has been in development for more than 10 years. The first attempt to remotely measure heart rate with ambient light was by Verkruysse et al. [7] in 2008, and measuring heart rate remains a challenge today. We applied a non-contact heart rate measurement method to an online teaching scenario; however, using the ECG and PPG methods to measure students’ heart rates in class has certain limitations in terms of scope of ease of use, cost, impact on subjects, etc. As a result of illumination variation, motion artifacts, and video compression, the cardiac pulses are small in comparison to the numerous other factors that affect skin appearance over time.

Heart rate detection based on face video largely depends on the illumination on the face. Both indoor and outdoor ambient lighting conditions are subject to constant change, and direct lighting sources can cast strong shadows that accentuate or diminish certain facial features [8]. There are two main methods to solve the illumination problems. The first method is to separate the illumination change signal from the pulsating signal using signal-processing methods such as the ensemble empirical mode decomposition (EEMD) algorithm [9,10]. The second method considers the facial region of interest (ROI) and background ROI to have similar illumination changes, and the background ROI is used as the noise reference to correct the interference of the illumination changes [11,12].

The distance (angle) from the light source to the skin tissue and to the camera is affected by motion artifacts. Poh et al. [13] proposed a blind source separation method to reduce the error caused by motion. In addition, there are some other methods to solve the problem of motion artifacts, such as Chrominance (CHROM) [14], wavelet transform [15], and bounded Kalman filter technology [16], etc.

Currently, most of the existing rPPG methods are effective based on uncompressed video data. McDuff et al. [17] pointed out that video compression has a great influence on video-based physiological signal extraction. The uncompressed videos occupy a large amount of storage space, which impedes the sharing of the data online. Zhao et al. [18] tried to extract the rPPG signal in the presence of video compression artifacts and proposed a video compression algorithm, named POSSC [19], so that the existing rPPG signal extraction methods can be directly applied to compressed video.

Although the aforementioned studies have made outstanding contributions to dealing with illumination variation, motion artifacts, and video compression, the video used for testing requires the whole frontal face to be included. However, situations in which the side of the face is directed to camera or the face is too close to the camera, are very common amongst students in class. As a result of the limitations of traditional non-contact heart rate detection, the above methods cannot work in the mentioned special cases. Thus, we improved it to apply to online teaching scenarios. In this paper, a method is presented to achieve heart rate detection under special conditions. In summary, our contributions are:

(1) We propose a symmetry substitution method. When the head is rotated 30 degrees to 45 degrees, and the region of interest in the face is partially missing, the data detected in the left and right cheeks are symmetrically copied;
(2) We designed a method to determine the effective facial region of interest (ROI) based on the face–eye location then calculate the physiological parameters;
(3) We designed a video dataset in minutes.

## 2. Related Work

The current research methods focus on illumination [16,20], motion [20,21,22], video compression [19], and other aspects, but less attention is paid to the selection of regions of interest, the impact of face detection methods, and the datasets suitable for heart rate detection.

The ROI of reliable sites is the key to extracting physiological parameters based on the rPPG method and directly affects the accuracy of the measured values [23]. Marnix et al. [24] found that the use of video cameras to collect facial skin tissues is very accurate in calculating heart rate through rPPG, but the measurement of heart rate in the wrist and calf region is not reliable. Therefore, all video-based heart rate measurements need to be recorded from the face. There are three commonly used regions of interest: the full face (the whole face detected using a face detection algorithm); three rectangular regions (the forehead and left and right cheeks); and the band region (a rectangular area of the left and right cheek and nose). Table 1 summarizes the regions of interest used in related articles and compares their effects.

Face detection is a crucial step in heart rate measurement. Histogram of Oriented Gradient (HOG) [29] is a local descriptor that uses a gradient vector direction histogram. It uses the pixel gradient distribution to describe the appearance and shape of objects. The Viola–Jones algorithm [30] describes the gray distribution of human faces using Harr features, improves the detection speed using an integral graph, and then constructs a cascade classifier using the AdaBoost algorithm. The multi-task convolutional neural network (MTCNN) model [31] contains three cascaded multi-task convolutional neural networks, which can detect face and feature points simultaneously. This model outperforms the state-of-the-art methods, and a small part of the face can be blocked during measurement. Deep Alignment Network (DAN) [32] is a cascaded depth neural network that employs a thermal map to provide visual information on the position of key points on the face. When a small part of the face is occluded, the DAN face detection model can also be carried out. This paper compares the advantages and disadvantages of these four face detection methods. Information regarding the detection effects is shown in Table 2.

In addition, most published papers regarding the recovery of HR from facial videos were assessed on privately owned datasets. However, it is not fair to compare different detection methods on different datasets. Therefore, a new publicly available dataset, directly related to rPPG-suitable practical applications, is vital. The currently available datasets include MAHNOB-HCI [33], DEAP [34], MMSE-HR [35], PURE [36], OBF [37], and VIPL-HR [38] et al., and their specifications are listed in Table 3.

At present, rPPG is tentatively applied in Intensive Care Units (ICU), because the subjects are still and a frontal face video can be continuously collected. Under these ideal conditions, the root mean square error (RMSE) of the traditional non-contact heart rate measurement method is between 1.24 and 8.90 bpm [16,27,39,40]. The frontal face RMSE of our proposed method reaches 3.49 bpm, which is very close to state-of-the-art traditional methods. However, it is rare that the face information is partly missing in medical scenarios. Therefore, the proposed method is more applicable in teaching scenarios.

## 3. Methods

### 3.1. Process

**Figure 1:**
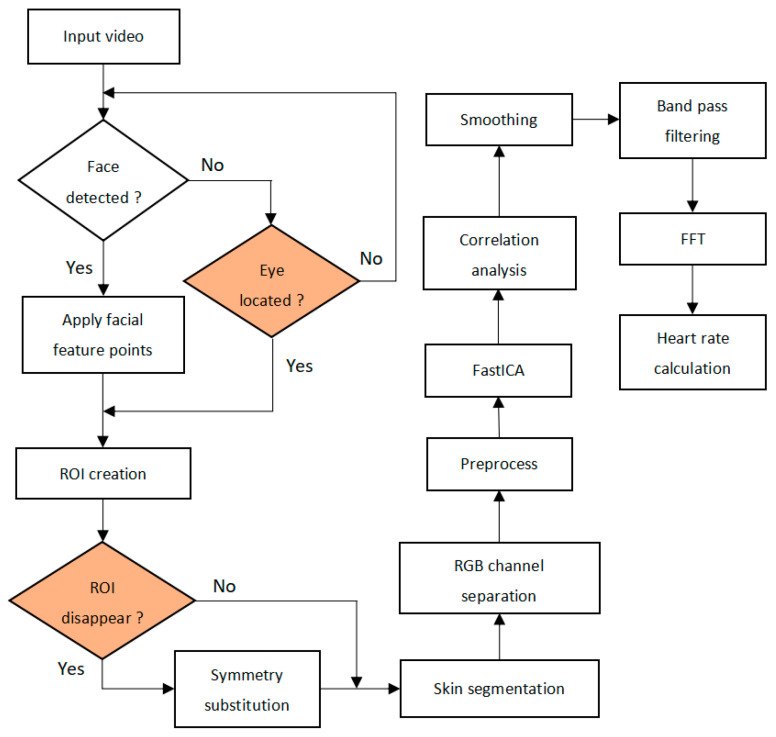
Outline of the proposed algorithm.

Traditional non-contact heart rate measurement process is divided into three steps. Firstly, the video frame is processed, then the Blood Volume Pulse (BVP) signal is extracted, and finally the physiological parameters are calculated. It is necessary for the face to be detected in all three steps to calculate the heart rate, but this is sometimes impossible. The objective of the method is to enable a real-time measurement of HR across different face position conditions. If the face is not detected, the eye region is located to find the region of interest, and then the heart rate is calculated. Figure 1 is an outline of the method. The following sections provide a detailed explanation of the various processes involved in achieving the objective. Our contributions are highlighted in color.

#### 3.1.1. Face Detection and Facial Feature Points Determination

In this step, face detection is carried out for each frame, and regions of interest are created based on the specific facial feature points on the detected face. With reference to Table 2, the MTCNN and DAN methods are accurate but time-consuming, and they are not suitable for real-time detection. The Viola–Jones face detection method is faster because it has no feature points marking function, and illumination intensity and direction do not easily change in online teaching conditions.

#### 3.1.2. ROI Creation

After face detection and feature point marking, it is necessary to select the ROI of face and extract the BVP signal related to physiological signal from the ROI. Regions of interest identification is a crucial step in heart rate measurement. On the basis of most methods, this paper discusses the influence of different face regions and their combinations on heart rate measurement. In our experiments, the face was divided into eight regions: left cheek; right cheek; forehead; left cheek and forehead; right cheek and forehead; left cheek and right cheek; left cheek and right cheek and forehead; left cheek and right cheek and nose. The heart rate values were calculated through these regions and compared with the ground truth. Then, the mean absolute error (MAE) was calculated for comparison to find the optimal region. Table 4 compares the different ROI definition methods and their respective measurement MAE. The optimal situation is expressed in bold.

Because there are more capillaries in the cheek area, the ROI from the cheeks and nose region was found to achieve better measurement results and hence were chosen as the ROI.

#### 3.1.3. Skin Segmentation

The principle of rPPG is to extract the signals related to the cardiac cycle from the subtle color changes in the skin. The background, clothes, teeth, hair, and other unrelated parts are useless for heart rate detection. Detecting such regions will not yield accurate HR measurements. Skin detection is performed on every frame to filter out non-skin pixels. This paper uses the skin color model established by Pitas et al. in *H*, *S*, *V* (Hue, Saturation, Value) space as given by Equation (1). It does not require color normalization and has strong robustness to illumination. Only when the following conditions are met can the skin be segmented:

(1) $$\{\begin{array}{c} \left(0^{o}\leq H\leq 25^{o}\right)\cup \left(335^{o}\leq H\leq 360^{o}\right)\\ \left(0.2\leq S\leq 0.6\right)\cap \left(0.4\leq V\right) \end{array}$$

**Figure 2:**
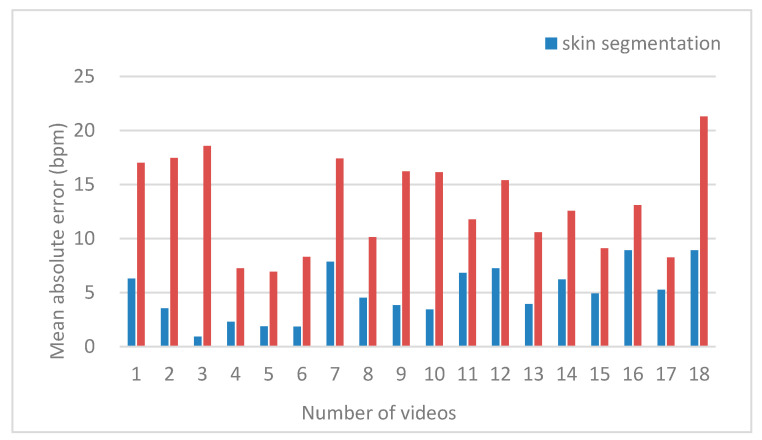
Comparison of the measurement effect between skin segmentation and non-skin segmentation.

The heart rate measurement effect of skin segmentation and non-skin segmentation was compared, and the calculation average error is shown in Figure 2. The results in Figure 2 illustrate that the heart rate measured by skin segmentation was closer to the ground truth, that is, the measurement accuracy can be improved by skin pixel extraction.

#### 3.1.4. Raw Traces Extraction and Signal Processing

**Figure 3:**
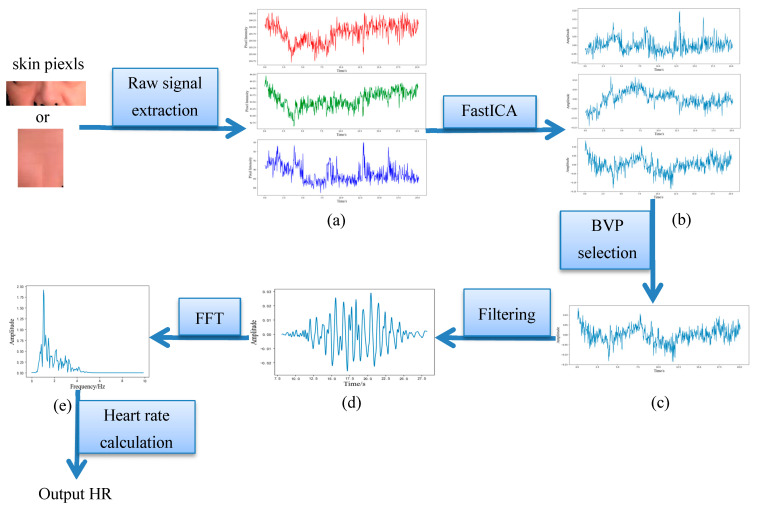
The detailed flowchart of our proposed method. (step a) Three one-dimensional signals. (step b) Three unsorted independent source components. (step c) The BVP signal. (step d) The filtered signal. (step e) The frequency spectrum of the refined BVP signal.

The raw red, green, blue (RGB) signals are obtained by calculating the average pixel value of the skin pixels within the ROI region over time. Then, the whole video sequence is transformed into three one-dimensional signals as raw signals, as shown in Figure 3 step a. After extracting the raw signals, signal-processing techniques such as detrending, normalization, smoothing, and filtering are employed to refine the signal. Firstly, we used detrending to remove linear trends from the raw signal, then the raw signal was normalized dividing the raw signal by its absolute value. Secondly, we employed a five-point sliding average filter to remove random noise.

#### 3.1.5. Independent Component Analysis

The RGB signals contain information about HR, but it is always mixed with noise. FastICA, one of the ICA methods, is an effective technique that can be utilized to eliminate noise artifacts. We used FastICA to extract the raw source signals from signals with noise. After FastICA, we obtained three unsorted independent source components from the extracted signals, as shown in Figure 3 step b. Verkruysse’s study showed that the green channel signal contains the strongest plethysmographic signal among all three channels [7]. Therefore, Pearson correlation analysis was performed between the three independent source signals and green channel signal. The Pearson correlation coefficients of the three components were 0.89, 0.46, and 0.04, respectively. This demonstrates a strong correlation between the upper example in Figure 3 step b and the green channel. Thus, the upper example in Figure 3 step b was selected as the BVP signal, as shown in Figure 3 step c. The other two components were discarded.

#### 3.1.6. Heart Rate Calculation

In this step, we applied a Hamming window-based bandpass filter with cutoff frequencies of 0.75 and 4 Hz to refine the BVP signal. This bandpass is common in previous research. The filtered signal is shown in Figure 3 step d. Then, we employed Fourier transform to obtain the frequency spectrum of the refined BVP signal, as shown in Figure 3 step e. The measured heart rate value can be calculated by finding the frequency corresponding to the highest peak value of the spectrum.

### 3.2. Symmetry Substitution Method

The subjects lose the left cheek or right cheek information in the region of interest when they face the camera sideways. On the basis of the statistical experiment (see Table 4 include some data), there was no significant difference in heart rate measurement between the left and right cheek. In this paper, a symmetrical substitution method is proposed, which replaces the undetected ROI of the left and right cheek with the one that can be detected. As a result of the limitations of the face detection algorithm, the face cannot be detected if the deflection angle is too large. Through experimentation, we found that the effective angle range of the symmetry substitution method proposed in this paper is 30 degrees to 45 degrees.

The goal of the symmetry substitution method is to calculate the *S*_left_ and *S*_right_ of the *ROI*_left_ and *ROI*_right_ (here, this refers to the left and right cheek in the video, contrary to the actual situation), and then judge whether there is a measurement missing through the area ratio. If it is missing, the symmetrical substitution method is used; if not, the original region of interest is used. Assuming that the area ratio of left cheek to right cheek is *a*, when *a* is greater than 1.5, the right cheek disappears; when *a* is less than 0.66, the left cheek disappears; when *a* is between 0.66 and 1.5, it indicates that no region of interest disappears as given by Equation (2).

(2) $$a=\frac{S_{left}}{S_{right}};\;ROI=\{\begin{array}{c} ROI_{nose}+2ROI_{left},a>1.5\\ ROI_{nose}+2ROI_{right},a<0.66\\ ROI_{nose}+ROI_{left}+ROI_{right},0.66\leq a\leq 1.5 \end{array}$$

### 3.3. Heart Rate Estimation by Face–Eye Location

**Figure 4:**
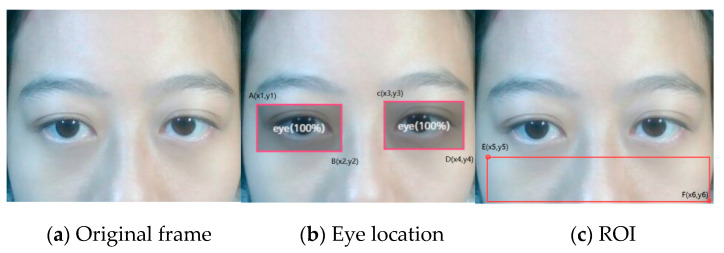
ROI location by eye while closing to camera.

**Figure 5:**
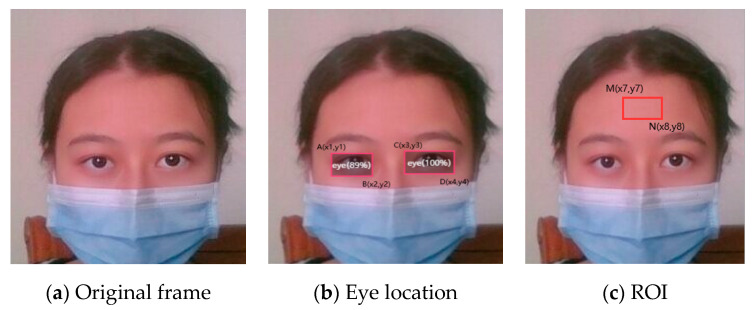
ROI location by eye while wearing a mask.

In the case of no face detection, the coordinates of human eyes are used in this paper to determine the region of interest in the face. The coordinate positions of the two eyes on the face are determined and marked with a rectangle, as shown in Figure 4b and Figure 5b. Suppose that the coordinates of point A of the left eye are (*x*_1_, *y*_1_), the coordinates of point B are (*x*_2_, *y*_2_), the coordinates of point C of the right eye are (*x*_3_, *y*_3_), and the coordinates of point D are (*x*_4_, *y*_4_). The position of facial ROI is determined by the coordinates of two eyes. When the subject’s face is too close to the camera, the coordinates of two points of E (*x*_5_, *y*_5_) and F(*x*_6_, *y*_6_) are those shown in Formula (3). When the subject is wearing a mask, the coordinates of two points of M (*x_7_*, *y_7_*) and N(*x_8_*, *y_8_*) are those shown in Formula (4).

(3) $$\{\begin{array}{c} x_{5}=x_{1}\\ y_{5}=y_{2}\\ x_{6}=x_{4}\\ y_{6}=y_{2}+\left(y_{2}-y_{1}\right)=2y_{2}-y_{1} \end{array}$$

According to the coordinates of the rectangle, the ROI region can be determined, as shown in Figure 4c and Figure 5c. The heart rate is further calculated according to the region. Because the height of the eye area is basically fixed each time, we found that the coordinate of *y*_5_ is equal to *y*_2_, the coordinates of *y*_6_ are equal to *y*_2_ plus the rectangle box height, *y*_7_ is obtained by subtracting three times the rectangle box height from the coordinates of *y*_2_, and *y*_8_ is obtained by subtracting one times the rectangle box height from the coordinates of *y*_3_. The size of the area enclosed by point M and N is suitable for the subsequent calculation.

(4) $$\left\{ \begin{array}{l} x_{7}=x_{2}\\ y_{7}=y_{2}-3(y_{2}-y_{1})=3y_{1}-2y_{2}\\ x_{8}=x_{3}\\ y_{8}=y_{3}-(y_{4}-y_{3})=2y_{3}-y_{4} \end{array}\right.$$

### 3.4. Participants

Subjects included three males and three females in our dataset. All subjects wore glasses or sunglasses. One of the female subjects covered her forehead and only showed her eyes and below. All subjects were asked to record videos without makeup.

### 3.5. Experimental Environment and Benchmark Dataset

**Figure 6:**
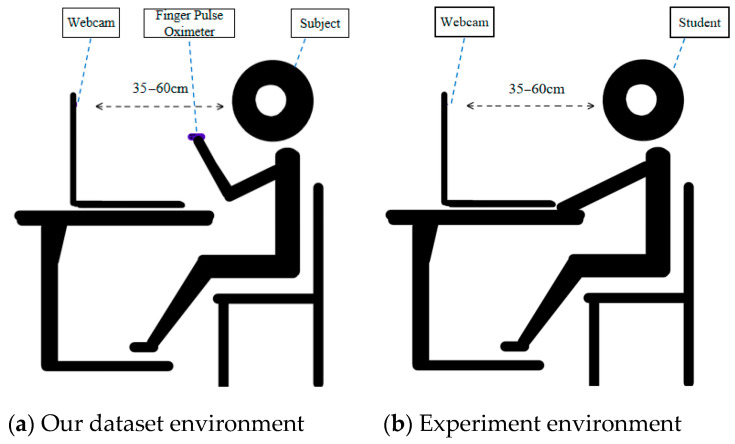
Dataset and experiment environment.

Several groups of videos were collected in two static states: head to camera and head side to camera. Videos were recorded in the natural environment at home. During the recording process, the subjects were required to hold the finger clip pulse oximeter (model DB12) for the real-time measurement. Various parameters are shown in Table 5. The measurement results can be displayed in the video, so that the recorded video contained not only facial information, but also the results of the detection using professional equipment (the real value), which was convenient for comparison with the calculated heart rate. Our dataset environment is shown in Figure 6a. The status of students in online class is shown in Figure 6b.

We created our own dataset containing 70 video sequences (each video lasting 60 s) using the Chicony USB 2.0 camera webcam. The light source was a mixture of fluorescent lamps and natural light. All videos were recorded in a 24-bit RGB color format with a resolution of 640 × 480, at 29.97 frames/s (NTSC video standard), and were stored as uncompressed data. When recording the video, the subjects were required to sit away from the camera (35~60 cm), keeping their head from shaking as much as possible. Their sight line was consistent with the camera level, and natural blinking was allowed. Our dataset description is shown in Table 6.

### 3.6. Emotion Experiments Design

Emotion can affect heart rate when the subject is stimulated [5]. We conducted two experiments using our method to prove that it can provide effective data support for emotion recognition. Ten students were subjected to emotional stimuli and asked to imitate emotions in the experiments.

The purpose of Experiment 1 was to verify that heart rate changes significantly when the emotion changes. We designed two scenarios in which the subjects could be angry while looking at a slide. When the subjects were watching the slide and their emotions changed, significant heart rate changes were detected by our method. Because the subjects watched at different speeds, the time it took to begin viewing certain scenes varied.

The purpose of Experiment 2 was to discover the heart rate changes when the subjects imitated the expressions, such as happiness, anger, surprise, etc., according to the cue.

## 4. Results

### 4.1. Results of the Symmetrical Substitution Method

This paper investigates the effects of two different ROI symmetric substitution methods, namely, the band region and the three rectangular regions.

**Figure 7:**
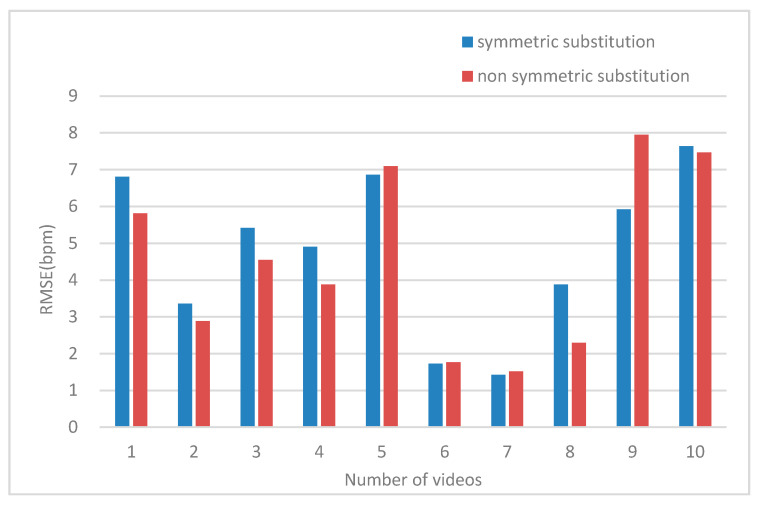
Comparison of the effect of the symmetrical substitution method in the band region.

For the band region, the comparison of the RMSE results between the symmetric substitution method and non-symmetric substitution method are shown in Figure 7.

Furthermore, we compared the effects of using the symmetric substitution method and not using the symmetric substitution method in the case of the frontal face. The symmetrical substitution method included two cases: replacing the right face with the left face and replacing the left face with the right face. The RMSE results are shown in Table 7. The optimal situation is expressed in bold.

**Figure 8:**
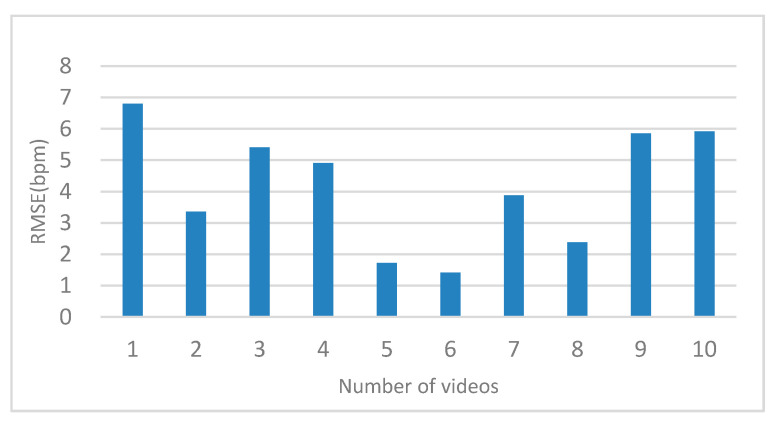
Comparison of the effect of the symmetrical substitution method in the three rectangular regions.

Because the three rectangular regions cannot estimate heart rate without a region of interest, only the symmetric substitution method can be considered. The RMSE is shown in Figure 8.

### 4.2. Results of Heart Rate Estimated by Face–Eye Location

When the whole face is not detected, the coordinates of the eyes are selected to determine the region of interest in the face. In the case of a frontal face, the method can detect human eyes whether the eyes are open or closed, and whether the eyes are looking out of the computer screen. In the case of a profile, if only one eye can be detected, the corresponding skin pixel under the eye is taken. The results of heart rate measurement with the face–eye location method are shown in Table 8. Although this allows for heart rate detection without facial detection, the detection time is increased.

### 4.3. Comparison with Other Methods

For special cases, we used the following methods on our database to compare with our method, and the results are shown in Table 9.

### 4.4. Results of Emotion Experiments

**Figure 9:**
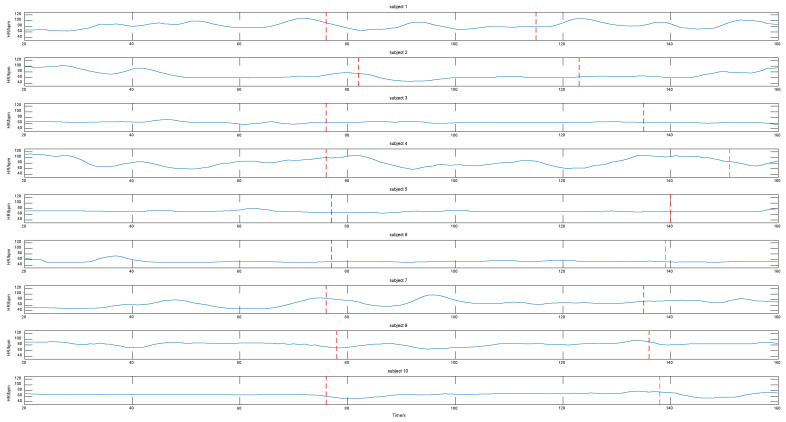
Heart rate changes when subjects were stimulated.

Firstly, the heart rate time series acquired using our method are illustrated in Figure 9. From 20 s to 160 s, there are two kinds of visual stimulation causing emotional changes in the subjects. According to the time point where the subjects switch slides, we marked two dotted lines in each graph. The section before the first dashed line represents the subjects before they were stimulated, and the section between the first and second dashed line represents the subjects seeing the scene that made them angry. The heart rates of subjects 1, 4, and 7 changed obviously. The heart rates of subjects 2, 9, and 10 changed slightly. The heart rates of subjects 3, 5, and 6 remained basically unchanged. The heart rate of subject 8 was undetected.

**Figure 10:**
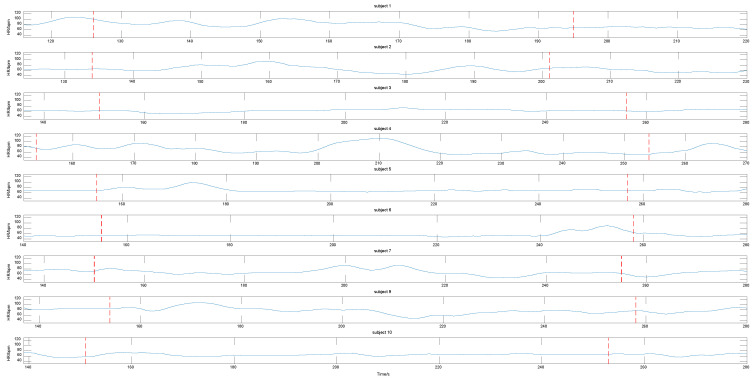
Heart rate changes when subjects imitated the expressions.

Secondly, we observed the changes in the heart rate of the subjects when they imitated expressions. The results are shown in Figure 10. The section of the subjects imitating the expressions was between the first and second dashed lines. The heart rates of subjects 3, 5, 6, and 10 remained basically unchanged. The heart rates of subjects 1, 7, and 9 were relatively stable in the second half of the imitation. The heart rates of subjects 2 and 4 changed obviously. The heart rate of subject 8 was undetected.

## 5. Discussion

On the basis of our study results, we illustrated the effectiveness of using the symmetrical substitution and face-eye location methods to estimate heart rate.

According to the definition of ROI in Table 4, the minimum average error of heart rate measured in each line appeared most frequently in the region of interest composed of the left and right cheek and nose. This is because the whole face area contains a mouth and eyes, and the signal-to-noise ratio is reduced due to unavoidable actions such as breathing and blinking. There are more capillaries in the cheek area, so the rPPG signal quality in this band region is the best. Compared with the average error measured by the left cheek or right cheek, it can be seen that there was no significant difference between them, which also provides a theoretical basis for symmetrical substitution.

When one cheek disappeared, the physiological signal was measured using the symmetrical substitution method. For the banded region of interest, we compared the ROI of skin pixels composed of the left and right cheek and nose with those obtained without the symmetrical substitution method. The RMSE, as shown in Figure 7, reveals that the same video measured using the symmetrical substitution method can be up to 2 bpm when the ROI disappears and the ROI is complete. We found that the RMSE of the 10 videos were distributed in the range of 1–8 bpm. We can conclude that the symmetrical substitution method does not have a significant influence on the measurement accuracy in the case of a profile. As shown in Table 7, we found that whether the left cheek is replaced by the right cheek or the right cheek is replaced by the left cheek, the measured heart rate results were similar to those without symmetrical replacement. For the three rectangular regions, when a region of interest disappears, the heart rate measurement cannot be carried out. In this case, the symmetrical substitution method is discussed. We found that the RMSE of the measurement was generally between 1–7 bpm as shown in Figure 8. Another result worth highlighting is that the darker the video, the higher the RMSE value.

Face–eye location can solve the problem of the face–camera distance being too small to detect the face. The ROI on the face under the eyes can be found through the coordinates, and then the heart rate can be estimated. Similarly, in the case of wearing a mask, if it is not possible to detect the face, our method locates the two eyes, then finds the ROI on the forehead, before extracting the skin pixels and detecting the heart rate. When the profile is positioned towards the camera, that is, the head is turned, our method can also locate a person’s eye, detect the skin area under the eye, and then detect the heart rate. Therefore, as long as the eyes are located, i.e., at least part of the forehead, left cheek, and right cheek are visible, the heart rate can be detected. It can be seen from Table 8 that the MAE and RMSE of the heart rate measurement value was less than 6.15 bpm, which indicates that the method of locating the ROI with human eyes is relatively accurate. When both the face and eyes could not be detected, we could detect the heart rate by detecting the skin pixels. However, cases in which face information could not be detected completely was not within the scope of the study. In our dataset, the videos were recorded with complete face information and partial face information.

From Figure 9 and Figure 10, we found that when subjects received stimulation, their heart rates increased, while simple imitation did not have a great impact on their heart rates. In online teaching, it is possible to analyze students’ real emotions through the change of students’ heart rates. Our method confirms that emotional changes can cause heart rate changes. However, there was no significant change in heart rate when the subjects imitated the expression according to the cue. The results show that our method can provide effective data support for emotion recognition.

Furthermore, in the absence of information, our RMSE was in a range of 4.79 to 6.15 bpm. Our accuracy was compared with other methods in special situations, as shown in the Table 9. Our method can estimate heart rate in the absence of information (while a mask is being worn, the face–camera distance is too small, or the profile is being presented), while traditional methods cannot estimate heart rate in these cases.

We use the value of non-contact heart rate detection to provide data support for changes in students’ emotion in online teaching scenarios. There is a lot of facial information missing in e-learning, which makes it impossible to measure heart rate with traditional methods based on rPPG. In the proposed method, we use the symmetrical substitution method to make up for the lack of ROI when the profile of the face is angled towards the camera, and use face–eye location instead of face location to solve the problem of masks and the face being too close to the camera, which are seldom mentioned by other researchers. The traditional methods cannot complete the heart rate detection in these situations.

From the analyses, we think that heart rate can be used as one of the reference indicators to detect real emotions, just like expressions. Therefore, our method can provide data support for learning state detection in smart education.

## 6. Conclusions

Using physiological signals measured using the rPPG method to evaluate students’ emotional problems in education and teaching has received a lot of attention in recent years. However, it is susceptible to problems when the head is rotated and the distance between the head and camera changes. Firstly, we presented a symmetrical substitution method. When the subjects’ head is rotated 30 degrees to 45 degrees, the detected ROI data are copied to the undetected areas. Our results indicate that the heart rate measurement accuracy does not decrease compared with that obtained in the full face condition. Secondly, this paper presents a method of heart rate detection based on face–eye location instead of face detection as in traditional methods. It can also detect heart rate under special circumstances, with a RMSE under 7.64 bpm. Thirdly, we found that the heart rate rises when the subjects receive stimulation, and the heart rate remains basically unchanged when they imitate expressions. In addition, the profile dataset contains videos recorded under special circumstances, providing data support for the research of physiological signal detection based on rPPG in real situations. Future research will be devoted to improving the real-time stability and accuracy of the method.

## Figures and Tables

**Table 1:** Comparison of different region of interest (ROI) definitions and measurement results.

Article	ROI Definition	Method	Result
[13]	full face	ICA	The root mean square error of the static dataset was 2.29 bpm, and that of moving dataset was 4.63 bpm.
[25]	full face	Project_ICA	In static, human–computer interaction and exercise recovery scenarios, the mean absolute deviation were 3.30, 3.93, and 9.80 bpm, respectively.
[16]	three rectangular regions	bounded Kalman filter	The average measurement error was 3 bpm when the subjects walked to the camera from 4 feet away.
[20]	three rectangular regions	RADICAL	The average error was 1.42 bpm in a well-controlled dataset.
[26]	three rectangular regions	PCA	The accuracy rate of heart rate measurement of five subjects was above 98%.
[27]	band region	EVM + CNN	74.13% of the test data were well estimated.
[28]	band region	ICA	The root mean square error was 2.258 bpm under static condition.

**Table 2:** Advantages and disadvantages of the four face detection methods.

	Face Detection Effect	Performance Description	Advantage	Disadvantage
HOG [29]	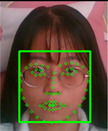	greatly outperforms the wavelet, PCA-SIFT, and Shape Context methods	fast running speed; 68 facial feature points	greatly influenced by light intensity and direction; inaccurate location of feature points on profile
Viola–Jones [30]	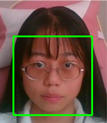	extremely rapid image processing, while achieving high detection rates	fast running speed	no facial feature point; greatly influenced by light intensity and direction
MTCNN [31]	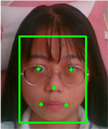	can achieve very fast speed in joint face detection and alignment	accurate face detection; less affected by light intensity and direction	complicated models; complex calculation; slow running speed; only five feature points can be marked
DAN [32]	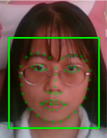	reduces the state-of-the-art failure rate by up to 70%	accurate location of feature points on profile; less affected by light intensity and direction; can mark 68 facial feature points	complicated models; complex calculation; slow running speed

**Table 3:** Available video datasets for physiological parameter detection.

Datasets	Number of Subjects	Number of Videos	Camera Parameters	Video Parameters
MAHNOB-HCI [33]	27	527	Allied Vision Stingray F-046C; F-046B	RGB videos 780 × 580 @ 60 fps
DEAP [34]	32	120	Sony DCR-HC27E	RGB videos 800 × 600 @ 50 fps
MMSE-HR [35]	40	102	RGB 2D color camera	RGB image sequences 1040 × 1392 @ 25 fps
PURE [36]	10	60	eco274CVGE	RGB videos 640 × 480 @ 30 fps
OBF [37]	106	2120	Blackmagic URFA mini	RGB:1920 × 1080 @ 60 fps
			Camera box	NIR:640 × 480 @ 30 fps
VIPL-HR [38]	107	3130	Logitech C310	960 × 720 @ 25 fps
			Realsense F200	NIR:640 × 480 @ 30 fps Color:1920 × 1080 @ 30 fps
			Huawei P9 smartphone	Color:1920 × 1080 @ 30 fps

**Table 4:** Comparison of different ROI definitions and measurement MAE (L = left cheek, R = right cheek, F = forehead, L-F = left cheek and forehead, R-F = right cheek and forehead, L-R = left cheek and right cheek, L-R-F = left cheek and right cheek and forehead, L-R-N = left cheek and right cheek and nose).

No.	L	R	F	L-F	R-F	L-R	L-R-F	L-R-N
1	12.73	12.38	10.28	16.35	14.13	12.05	16.68	**8.15**
2	8.75	6.15	8.50	7.85	7.08	10.75	6.38	**5.50**
3	8.13	9.95	7.95	7.25	8.33	8.85	6.18	**5.68**
4	8.46	**6.10**	8.05	9.41	7.21	11.41	8.36	6.97
5	2.93	1.33	3.69	3.36	3.55	2.36	**1.29**	1.48
6	1.57	1.95	**1.50**	3.19	2.88	1.90	2.57	3.12
7	4.36	0.69	4.38	3.81	2.36	1.86	3.74	**0.38**
8	5.93	1.34	18.10	12.49	15.71	**1.27**	15.54	1.39
9	23.40	11.10	18.52	21.36	16.17	4.57	16.33	**2.86**
10	9.81	4.79	7.95	3.67	**3.14**	3.29	3.88	4.81

**Table 5:** DB12 parameters.

Parameter	Value
Pulse Rate Display	25–250 bpm
Resolution	1 bpm
Measurement accuracy	2 bpm

**Table 6:** Dataset description.

	Classification	Distance (cm)	Numbers
Frontal face	with sunglasses	35–60	10
	Wearing a mask	35–60	10
	too small a face–camera distance	<20	10
	no occlusion of the face	35–60	30
Profile	no occlusion of the face	35–60	10

**Table 7:** Comparison of the effect of the symmetrical substitution method in the frontal face (L-R = replace right cheek with left cheek; R-L = replace left cheek with right cheek; N-U-S = No use of symmetrical substitution).

Methods	1	2	3	4	5	6	7	8	9	10
L-R	5.53	**5.42**	**2.57**	2.15	3.98	2.44	5.43	5.99	3.69	**0.99**
R-L	**1.32**	5.43	4.69	**1.37**	**3.62**	3.78	**1.76**	**2.36**	**1.25**	1.47
N-U-S	2.25	6.86	3.77	1.41	5.20	**1.75**	3.81	2.77	2.36	1.88

**Table 8:** Heart rate measurement results from the face–eye location method.

Video	MAE (bpm)	RMSE (bpm)
Too small a face-camera distance	4.00	5.87
Wearing a mask	4.19	6.15

**Table 9:** Comparison of heart rate measured by different methods.

Methods		RMSE in Different Conditions (bpm)			
		Frontal Face	Profile	Too Small a Face-Camera Distance	Wearing a Mask
CHROM_De Haan [14]	Viola–Jones [30]	1.1	Undetected	Undetected	Undetected
	Skin segmentation	1.44	10.51	9.45	21.34
ICA_Poh [39]	Viola–Jones [30]	1.24	Undetected	Undetected	Undetected
	Skin segmentation	6.53	11.18	30.25	26.20
POS_Wang [41]	OC-SVM [42]	8.04	Undetected	Undetected	Undetected
	Skin segmentation	8.37	20.74	13.61	24.69
Bounded Kalman filter [16]	Viola–Jones [30]	5.56	Undetected	Undetected	Undetected
	Back Projection	6.62	Undetected	Undetected	Undetected
Proposed algorithm		3.49	4.79	5.87	6.15
